# Highly Selective Removal of Cationic Dyes from Wastewater by MgO Nanorods

**DOI:** 10.3390/nano12061023

**Published:** 2022-03-21

**Authors:** Monira Galal Ghoniem, Fatima Adam Mohamed Ali, Babiker Yagoub Abdulkhair, Mohamed Rahmt Allah Elamin, Arwa Mofareh Alqahtani, Seyfeddine Rahali, Mohamed Ali Ben Aissa

**Affiliations:** 1 Department of Chemistry, College of Science, Imam Mohammad Ibn Saud Islamic University (IMSIU), Riyadh 11432, Saudi Arabia; mgghoniem@imamu.edu.sa (M.G.G.); babiker35.by@gmail.com (B.Y.A.); mohamedrahmt99@gmail.com (M.R.A.E.); amalqhtani@imamu.edu.sa (A.M.A.); 2 Department of Chemistry, College of Science and Arts, Qassim University, Ar Rass, Saudi Arabia

**Keywords:** MgO nanomaterials, basic fuchsine elimination, pH influence, kinetics experiment

## Abstract

The organic synthetic dyes employed in industries are carcinogenic and harmful. Dyes must be removed from wastewater to limit or eliminate their presence before dumping into the natural environment. The current study aims to investigate the use of MgO nanoparticles to eliminate basic fuchsine (BF), as a model cationic dye pollutant, from wastewater. The MgO nanorods were synthesized through a coprecipitation method. The obtained nanocomposite was characterized using various techniques such as X-ray diffraction (XRD), field emission scanning electron microscopy (FE-SEM), Brunauer–Emmett–Teller (BET), and FTIR spectroscopy. It was found that the variation of dye concentration and pH influenced the removal of BF by MgO. The adsorption capacity of 493.90 mg/g is achieved under optimum operating conditions (pH = 11, contact time = 236 min, and initial BF concentration = 200 ppm). Pseudo-second-order adsorption kinetics and Freundlich isotherm models best fitted BF sorption onto MgO nanorods. The BF sorption mechanism is associated with the electrostatic attractions and hydrogen bond between the O–H group of MgO and the NH_2_ groups of BF, as indicated by the pH, isotherms, and FTIR studies. The reusability study indicates that MgO was effectively used to eliminate BF in at least four continuous cycles. The investigation of MgO with different dyes suggests the high adsorption selectivity of BF, crystal violet (CV), and malachite green (MG) dyes compared with methyl orange (MO) dye. Overall, MgO nanorods can act as a potential and promising adsorbent for the efficient and rapid removal of cationic dyes (CV, MG, and BF) from wastewater.

## 1. Introduction

**Figure 1:**
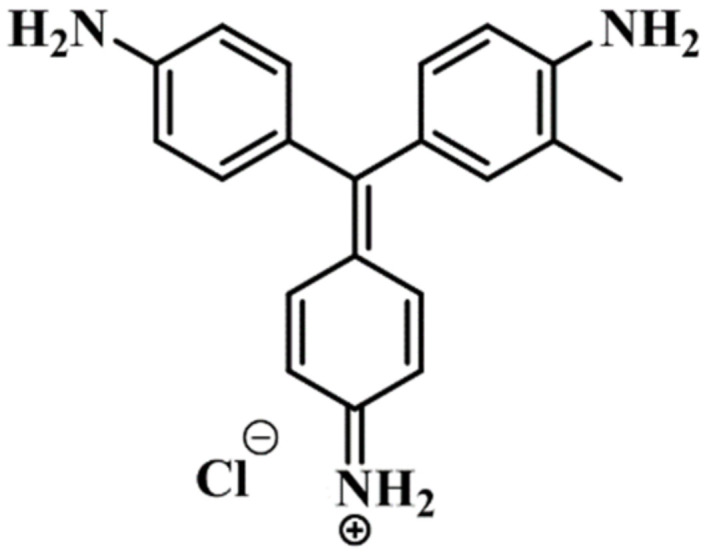
Chemical structure of basic fuchsine (BF).

Organic synthetic dyes are extensively used in various industries, including in printing, textiles, paints, and pigments [1]. The discharge of dye effluents from various industries has posed a grave hazard to the marine environment and human health. Additionally, most dyes are extremely poisonous and can have mutagenic, carcinogenic, and teratogenic impacts on humans and aquatic life at extremely low concentrations [2]. Cationic dyes have been reported to be more toxic than anionic dyes, with tinctorial values of 1 mg/L [3]. Basic fuchsine (BF) is a cationic dye utilized in diverse fields, including biological stains and leather, paper, and cotton coloring agents [4,5]. Additionally, it is referred to as basic violet or rosaniline chloride [6]. It is a member of a triphenylmethane dye class with the chemical formula C_20_H_20_ClN_3_ [7] and boasts an impressive planar conjugated π structure (Figure 1) [8]. Despite this, the basic fuchsine (BF) dye is combustible, anesthetic, and bacteriostatic [9,10]. It has been classified as a probable carcinogen in several nations because of its toxicity, carcinogenicity, and poor biodegradability [11,12]. As a result, much effort has been devoted to removing BF from wastewater. A variety of physical, chemical, and biological strategies have been employed to remove dye pollutants from environmental wastewater, including biodegradation [13], chemical oxidation [14], ion exchange [15,16], membrane filtration [17], and adsorption [18,19,20]. Even though photocatalysis is a simple, highly efficient, and advantageous technique for removing dye contaminants [21], it has some drawbacks. It is not a cost-effective approach, photocatalysts leach into the water system, and it is ineffective in dark conditions [22]. The adsorption method is considered the most effective technology for removing dye pollutants from water due to its low cost, high efficiency, versatility, and ease of use [23]. The critical component of this approach is the development of high-performance adsorbents. Numerous studies have been conducted to explore nanomaterials as efficacious sorbents for the elimination of cationic dyes from used water [24,25]. Several nanomaterials, including Y_2_O_3_@ZnO [18], TiO_2_/MWCNTs [20], and starch-capped zinc selenide [26], have been investigated as adsorbents for BF elimination from wastewater.

Magnesium oxide (MgO) exhibits excellent surface characteristics at the nanoscale due to its polyhedral structure and the formation of Frenkel or Schottky defects at the edge/corner [27]. These features result in a high surface area for the nanomaterials, making them applicable for environmental remediation.

The current study aims to investigate the manufacturing of MgO nanorods and evaluate their adsorption ability to remove cationic dyes. The MgO characteristics were studied using SEM, BET, XRD, and FTIR. The effectiveness of the elimination parameters such as contact time, initial BF concentration, and pH toward the sorption capability of MgO nanorods was examined. Additionally, kinetic studies, adsorption isotherms, and reusability were also studied. The plausible mechanism of BF dye removal by MgO nanoparticles was scrutinized by FTIR and pH studies.

## 2. Experimental

### 2.1. Chemicals

Magnesium nitrate (≥99.0%), ammonia solution (NH_4_OH, 25%), basic fuchsine (BF, ≥85%), malachite green (MG, ≥90%), crystal violet (CV, ≥90%), methyl orange (MO, ≥85%), sodium hydroxide (NaOH, ≥99%), sodium chloride (NaCl, ≥99%), and hydrochloric acid (HCl, 37%) purchased from Merck Company were used without further purification. The required concentrations (25 to 300 ppm) were obtained by diluting the BF stock solution (500 ppm).

### 2.2. Preparation of MgO Nanorods

Magnesium oxide nanorods were synthesized in two steps: magnesium hydroxide precipitation and then calcination at 700 °C until MgO formation. Magnesium hydroxide Mg(OH)_2_ was obtained by adding increments of aqueous ammonia (NH_4_OH) to 200 mL of magnesium nitrate solution (0.1 M) until precipitation and keeping constant stirring using a magnetic stirrer for 12 h. The precipitate was washed, dried, and calcined using a combustion furnace at 700 °C for about 2 h [28].

### 2.3. Characterization of MgO Nanorods

Field emission scanning electron microscopy (FE-SEM) was used to study the morphology of the as-prepared MgO nanorods using a high-resolution JEOL JEM-6700F apparatus integrated with electron dispersive X-ray spectroscopy (EDS) for elemental chemical composition determination. X-ray diffraction (XRD) was used to determine the crystal structure and phase purity using a Rigaku Mini Flex 600 (Tokyo, Japan) diffractometer equipped with a CuK radiation source (λ = 1.5417 Å). The Brunauer–Emmett–Teller (BET) formula and Lippens and de Boer’s t-plot approach were used to determine the surface area and porosity of MgO. The vibration modes of MgO nanorods were determined before and following BF dye adsorption using a JASCO FT-IR spectrometer.

### 2.4. Dye Adsorption Experiment

Batch elimination investigations were carried out by putting 10 mg of MgO nanorods in 25 mL of dye solution with varying beginning concentrations C_0_ (25–300 ppm) on a magnetic stirrer for 24 h. Following each experiment, the samples were centrifuged, and the dye concentrations were concluded using a Shimadzu spectrophotometer (UV-1650 PC). The remaining dye concentration was obtained at a maximum wavelength of 545 for BF, 590 nm for CV, 579 nm for MG, and 465 nm for MO. The spectrophotometer was used to estimate the dye concentrations C_t_ at t time and Ce at the equilibrium. The following equation was used to determine the organic dye capacity (q_e_):

(1) $$q_{e}=\left(C_{0}-C_{e}\right)\frac{V}{m}$$

For the kinetic study, the mass of MgO (m), the initial concentration, and the volume of BF solutions were 60 mg, 200 ppm, and 150 mL (V), respectively. At fixed intervals of time, 5 mL of the mixture was retired and centrifuged for determining the residual BF concentration [29]:

(2) $$q_{t}=\left(C_{0}-C_{t}\right)\frac{V}{m}$$

The BF sorption on the as-prepared MgO was investigated, the adsorption capacity (q_t_) and percentage (Ads %) were calculated utilizing Equations (3) and (4), respectively.

(3) $$q_{t}=\frac{\left(C_{o}-{\;C}_{t}\right)\;V}{M}\;$$

(4) $$\mathrm{Ads}\;\%=\frac{\left(C_{o}-{\;C}_{t}\right)}{C_{o}}\times 100$$

C_o_ (mg L^−1^) and C_t_ (mg L^−1^) represent initial and unadsorbed concentrations; M (kg) and V (L) represent the solution’s volume and sorbent’s mass. Remarkable fast uptake and high experimental q_t_ are the most important properties for excellent sorbents. In our case, both conditions were fulfilled as the prepared MgO possessed a practical adsorption capacity of 496 mg/g.

## 3. Results and Discussion

### 3.1. MgO Characterizations

**Figure 2:**
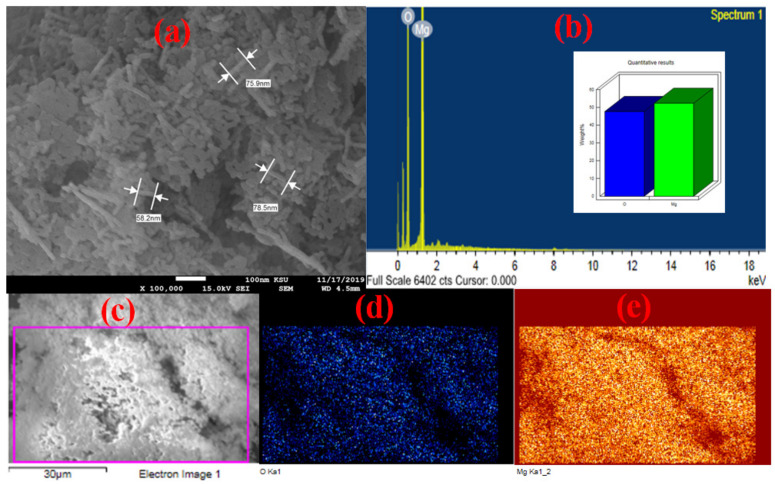
(**a**) SEM image and (**b**) EDX spectrum of MgO; X-ray elemental mapping of (**d**) Mg−Ka and (**e**) O−Ka of the SEM selected area (**c**).

To illustrate the structural and morphological properties of the nanomaterial as-synthesized, SEM micrographs of the obtained MgO nanomaterials are shown in Figure 2a. MgO appears to have a rod-like structure. The elemental composition and elemental mapping of the produced MgO were determined using EDS in conjunction with SEM (Figure 2b). In addition to the oxygen peak, the EDS analysis of MgO revealed the presence of a powerful and sharp magnesium peak without any impurity peak. The elemental mapping of the produced nanomaterials revealed that Mg and O were distributed uniformly within the MgO (Figure 2d,e).

**Figure 3:**
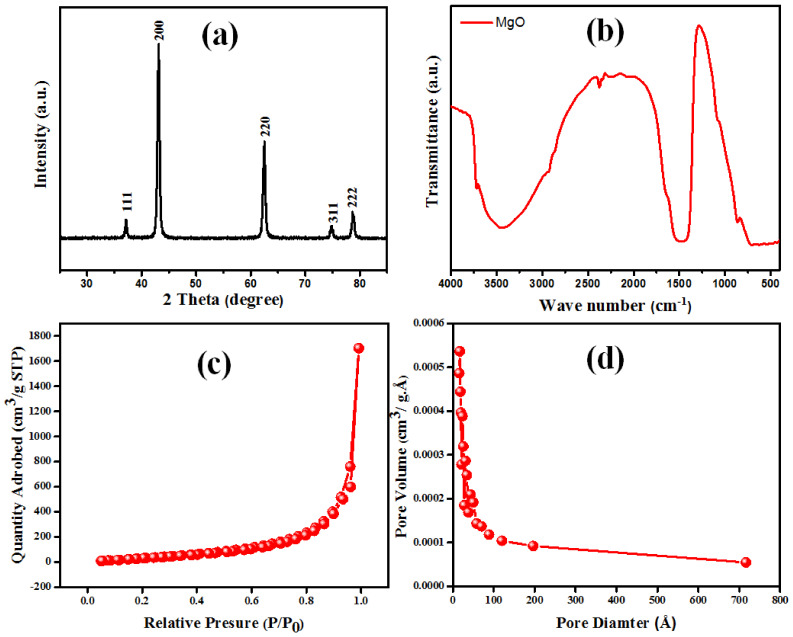
(**a**) XRD patterns, (**b**) FTIR spectra, (**c**) pore size distribution, and (**d**) nitrogen adsorption–desorption isotherm of MgO nanorods.

The XRD pattern of the as-obtained MgO nanorods is illustrated in Figure 3a. The main diffraction peaks at 2θ = 78.7, 74.8, 62.3, 43.0, and 37.1° could be indexed to the (222), (311), (220), (200), and (111) planes of MgO structure (JCPDS 87-0653) [30]. No impurity peaks were found, indicating that the MgO nanorods formed were pure.

Figure 3b illustrates the FTIR spectra of the as-fabricated MgO nanorods. The sharp and strong band at 3725 cm^−1^ was assigned to the A_2u_(OH) lattice vibration [31]. The large band at 3438 cm^−1^ was assigned to the stretching vibration of the O–H group [32]. Bands in the range 1458–1640 cm^−1^ were linked to the adsorbed water molecule’s −OH stretching mode. It is widely recognized that MgO can react with water to generate Mg(OH)_2_ [33]. The characteristic bond occurred at 820 cm^−1^, confirming the existence of MgO bands [32]. The pore size distribution and N_2_ adsorption–desorption isotherms of the as-obtained MgO nanoparticles are exhibited in Figure 3c,d, respectively. The results indicate that the isotherm is a type IV hysteresis buckle with H3 hysteresis buckle [34]. The MgO nanoparticles’ average pore diameter and BET surface area are 1.7 nm and 12.215 m^2^/g, respectively.

### 3.2. Adsorption of BF

#### 3.2.1. Influence of Dye Concentration and pH on BF Adsorption

**Figure 4:**
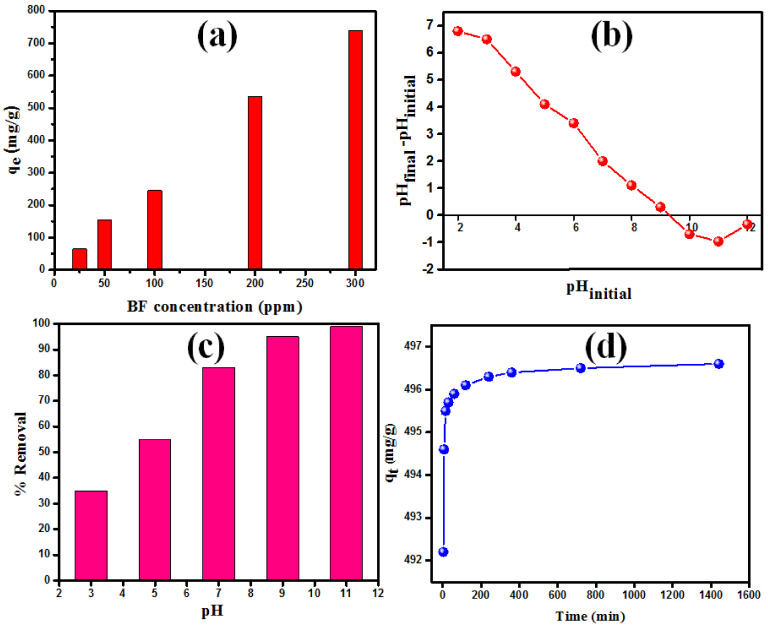
(**a**) Effect of initial BF dye concentration on the adsorption on MgO at pH = 7, (**b**) plot for the determination of pHZPC of MgO, (**c**) the influence of pH on % elimination of BF with an initial concentration of 200 ppm, and (**d**) equilibrium time study with a BF starting concentration of 150 ppm.

The studies on the influence of dye concentration were performed at pH = 7 using varied BF concentrations (from 25 to 300 ppm). The adsorption experiments were conducted employing 10 mg of MgO nanoparticles and 25 mL of BF solution. Figure 4a shows that the adsorbed amount of BF increases from 65.45 to 738.79 mg/g when the initial BF concentration increases. When the initial BF concentration increases, the effective driving force generated overcomes any resistance of BF migration from the solution [35,36].

Many studies prove that the solution pH value plays a crucial role in the adsorption process by modifying the surface charge of nanomaterials [23,37]. Therefore, the surface is positively charged at pH lower than pHZPC value and negatively charged at pH greater than pHZPC value. The MgO’s pHZPC was obtained using the solid addition method. [38]. Starting with a solution of KNO_3_ (0.1 M), the pH of the solution was adjusted from 3 to 11 by gradually adding HCl or NaOH solutions. Then, 10 mg of adsorbent was added to 10 mL of pH-adjusted KNO_3_ solution (pH_i_) and stirred for one hour. After that, the final solution pH (pH_f_) was measured, and the difference between the pHf and pHi was plotted versus the pH_i_ (Figure 4b). The point where pH_f_ − pHi = 0 was designated the pHpzc equal to 9.2 for MgO (Figure 4b). To explain the effect of the solution pH value on the BF dye elimination performance, the pH experiment was carried out employing 10 mg of MgO with an initial BF concentration of 200 ppm that was regulated at 3, 5, 7, 9, and 11 pH values. The pH’s effect on BF elimination performance is shown in Figure 4c. It can be remarked that low adsorption efficiency was obtained at acidic pH values, while higher elimination efficacy of BF was attained at basic pH values. The low adsorption efficiency obtained at lower pH values is due to electrostatic repulsion between the MgO sorbent and the BF dye [39]. The electrostatic attraction between MgO and BF molecules improves the dye adsorption at basic pH [40].

#### 3.2.2. Kinetics of BF Adsorption on MgO Nanorods

**Figure 5:**
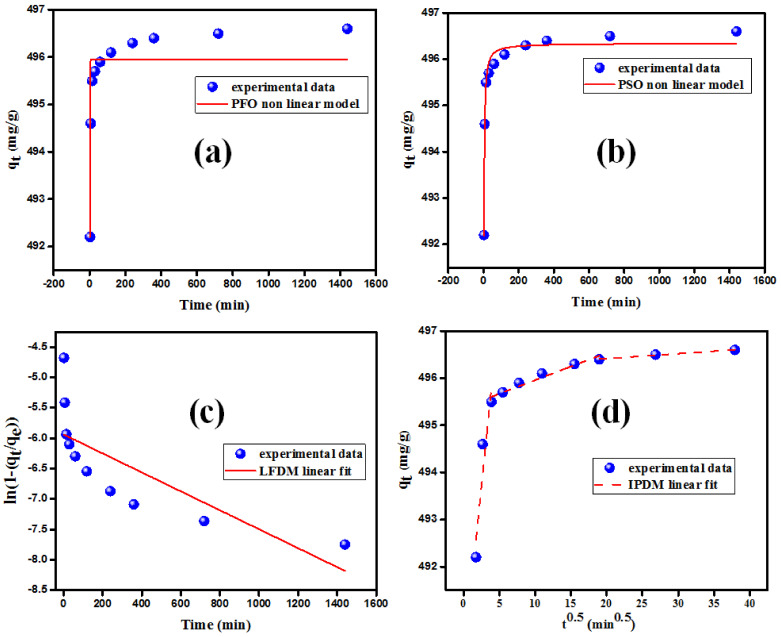
(**a**,**b**) Nonlinear pseudo-first-order and pseudo-second-order kinetic plots; (**c**,**d**) the liquid film diffusion model and intraparticle diffusion model plots.

At room temperature, the effect of agitation time on the adsorption of BF molecules onto MgO nanorods was investigated by altering the agitation period between 5 and 1440 min with a BF starting concentration of 150 ppm (see Figure 4d). The rate of BF elimination increases steadily as the contact time increases and achieves equilibrium within 236 min. At the start of the procedure, the adsorption rate was extremely rapid due to the accessibility of a high number of active sites on the surface of MgO nanorods. The equilibrium concentration of the remaining active sites is lowered, and the sorption rate becomes extremely low. As a result, the elimination of BF molecules is unaffected. The nonlinear formulas of the adsorption kinetic were utilized to avoid spurious findings of the linearized equations [41,42]. Hence, the original equations of pseudo-first-order (PFO) and pseudo-second-order (PSO) (Table 1) were employed [43,44,45]. k_1_ (min^−1^) and k_2_ (g mg^−1^ min^−1^) presented the rate constants for PFO and PSO, respectively. The k_1_ and k_2_ values were extracted from the obtained slope values of the PFO and PSO plots (Figure 5) [46].

The best-fitted kinetic model was selected according to the correlation coefficient values (R^2^) and the q_t_ values calculated from the regression [41]. The obtained results indicated that BF adsorption on MgO nanoparticles followed the PSO with an estimated qt value similar to the experimental one (Table 1 and Figure 5).

The preliminary data analysis established that the PSO kinetic model is suitable for modeling the adsorption of BF onto MgO nanorods. This includes the entire adsorption process involving liquid film diffusion (LFDM), adsorption on the adsorbent surface, and intraparticle diffusion (IPDM) [49,50]. Due to the accelerated rate, the general adsorption response cannot be regarded as a controlling step in the adsorption process. Thus, the rate of adsorption was mostly determined by slower stages such as LFDM and IPDM, either alone or together. The equations of intraparticle (IPDM) [48] and liquid-film diffusion models (LFDM) [51] are presented in Table 1. K_LF_ (min^−1^) and the K_dif_ (mg/g min^−0.5^) are LFDM and IPDM factors. C_i_ (mg/g) is a factor that represents a boundary layer thickness. The linear regression plots for LFDM and IPDM are illustrated in Figure 5, and the results extracted from the graphs are displayed in Table 1. The BF sorption on MgO appeared to be controlled by the IPDM, as determined by the R^2^ values, and three linear segments can be seen when piecewise linear regression is used. In addition, the IPDM fitting curve did not pass through the origin point, implying that the primary rate-controlling mechanism is intraparticle diffusion [50].

#### 3.2.3. Adsorption Equilibrium

One of the most significant characteristics of adsorbents is the uptake capacity. Here, this is the maximum quantity (q_max_) of BF that MgO can remove. To calculate the uptake capacity of MgO nanoparticles, the adsorption results were adopted for the most used adsorption isotherms: Temkin, Dubinin–Radushkevich, Freundlich, and Langmuir. The formulas of the employed isotherm models are provided in Table 2.

**Figure 6:**
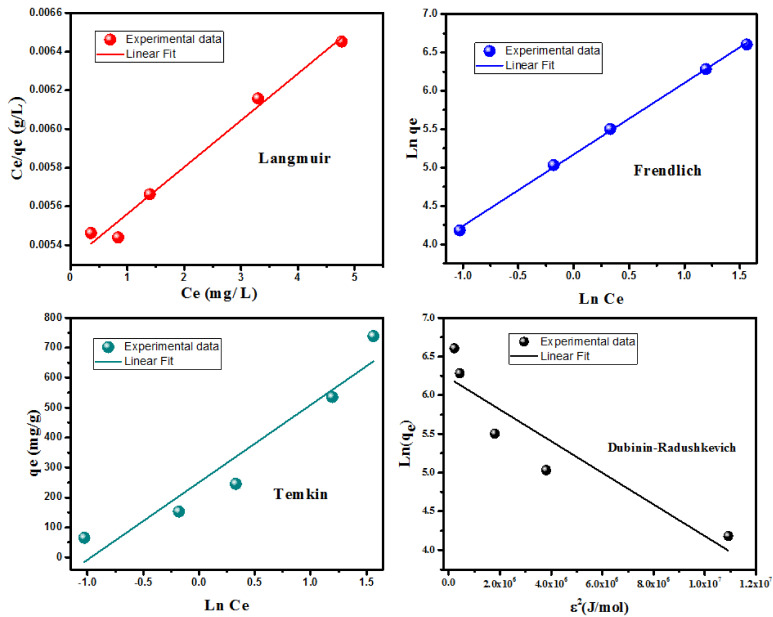
Adsorption of BF equilibrium models.

The linear isotherm graphs for BF adsorption on MgO are shown in Figure 6, and the characteristics of the adsorption isotherms are listed in Table 2. As demonstrated in the obtained results (Table 2), the Freundlich model for BF adsorption provides the best match with the greatest regression coefficient (R^2^ = 0.9957). The great value of the exponent n = 2.07, in the gamut 2–10, indicates superior adsorption properties [56]. In the Dubinin–Radushkevich isothermal model, the E value ((2K)^0.5^) indicates whether the adsorption process is mainly chemical or physical [57]. The mean energy value (E) was calculated as 6.38 kJ/mol. The obtained energy value is less than 8 kJ/mol, confirming that the BF adsorption on MgO is a physisorption process [58,59].

MgO’s adsorption capacity for BF is 493.9 mg/g, according to the Langmuir isotherm model, as shown in Table 2. It is advantageous to compare the MgO adsorption capacity with several BF dye adsorbents. Table 3 reveals the various adsorption capacities of sorbents for BF removal when compared to MgO nanorods. It is noteworthy that the MgO has a higher adsorption capacity than previously reported sorbents. Indeed, under the same pH condition (pH = 12), Tabrez A. Khan et al. studied the removal of basic dyes from aqueous solution by adsorption onto binary iron–manganese oxide coated kaolinite [60]. They found that the Fe-MgO/kaolinite adsorption capacity is 10.36 mg/g, which is very low compared to that found in our study. Likewise, Bouchra Ba Mohammed et al. successfully synthesized the Fe-ZSM-5 zeolite for BF dye adsorption from aqueous solutions [61]. The maximum uptake capacity was found to be 251.87 mg/g, which is lower than that of MgO. This finding established the efficacy of MgO nanorods as a good BF adsorbent.

### 3.3. Adsorption Mechanism

**Figure 7:**
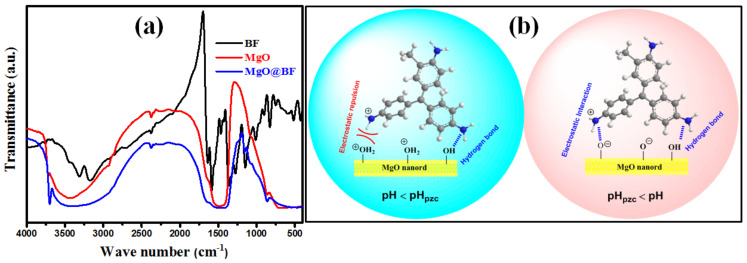
(**a**) FTIR spectra of MgO, BF, and MgO@BF and (**b**) the proposed adsorption mechanism of BF onto MgO nanorods.

FTIR study was employed to explain BF dyes’ selectivity and adsorption mechanism onto MgO nanorods. FTIR measurements were accomplished for the obtained MgO nanoparticles, Bf dye, and MgO nanoparticles after adsorption. Figure 7a presents the FTIR spectra of BF, MgO, and BF@MgO. The BF spectrum exhibits several distinct bands, including NH_2_ bending (3314 and 3175 cm^−1^), C=N stretching (1638 and 1325 cm^−1^), and aryl CH wagging (907–831 cm^−1^). The MgO spectrum exhibits the absorption band at 3461 cm^−1^ attributed to the O–H stretching vibration group that demonstrates the existence of hydroxyl groups in the MgO nanoparticles. After adsorption, the O–H group of MgO interacts with the lone-pair electrons of NH_2_ groups of BF via hydrogen bonds, which is confirmed by the slight shifting of the O–H stretching vibration band to 3384 cm^−1^. The adsorption effectiveness at lower pH levels, despite the electrostatic attraction between the MgO sorbent and the BF dye, demonstrates the presence of hydrogen bonding between the BF and MgO nanoparticles. In addition, the MgO@BF spectrum exhibits the presence of the characteristic BF in shifted position relative to the free molecules, showing the presence of MgO and BF interactions. In that order, the new band at 1671 cm^−1^ in the MgO@BF spectrum indicated the formation of ionic interactions between the BF molecules and the negative-charged MgO surface [65]. Following the pH, the study implies that the electrostatic attraction controls the BF sorption mechanism onto the MgO sorbent. The recommended BF sorption mechanism onto the MgO involves electrostatic attractions and hydrogen bonds. The recommended adsorption mechanism of the BF onto MgO nanorods is presented in Figure 7b.

### 3.4. Reusability and Selectivity Study

**Figure 8:**
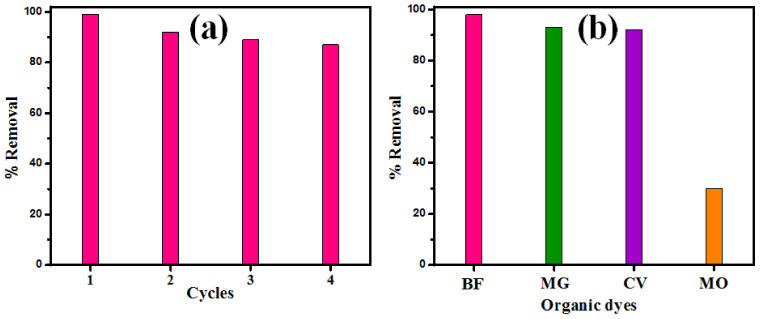
(**a**) Reusability efficiency of MgO NPs and (**b**) the elimination rate of different dyes by MgO adsorbent.

The reusability and the regeneration of MgO sorbent were examined by BF elimination from the surface of the nanomaterial. After the adsorption experiment, the dye desorption from the MgO was conducted by heating in an oven at 500 °C for 60 min. After that, the recuperated MgO was reused for the BF elimination. The reusability results indicate that MgO was effectively used to eliminate BF in at least four continuous cycles. (Figure 8a).

The high adsorption capability of MgO nanorods towards BF dye has also been examined compared to other organic dyes (BF, CV, MG, and MO). The selectivity study of MgO nanoparticles was examined at pH = 7 using fixed concentrations (50 mg L^−1^) f different dyes solutions, namely basic fuchsine (BF), crystal violet (CV), malachite green (MG), and methyl orange (MO). After adsorption, the remaining dye concentration was obtained at a maximum wavelength of 545 for BF, 590 nm for CV, 579 nm for MG, and 465 nm for MO. Figure 8b presents the removal percentages of different dyes eliminated by MgO nanoparticles. Outcomes show that the elimination ability percentages of BF, CV, MG, and MO were 98%, 93%, 92%, and 30%, respectively. However, BF, CV, and MG dye uptakes were higher than MO dye uptake. The investigation of MgO nanorods with different dyes suggests the high adsorption selectivity towards cationic dyes (BF, CV, and MG) compared with MO dye.

## 4. Conclusions

The precipitation method was successfully employed to fabricate MgO nanorods. The obtained results displayed an excellent elimination efficiency toward BF dye. The impact of BF concentration and pH were scrutinized to improve the removal performance of the MgO nanorods. Batch experimental studies revealed that the BF dye removal onto MgO nanorods was pH-dependent with a maximum adsorption capacity at pH = 11 for BF pollutant, i.e., 493.90 mg/g. The removal of BF by MgO nanorods was examined using different kinetic and adsorption models, and the best fit was provided by the PSO kinetics and Freundlich adsorption isotherm model. The BF adsorption mechanism was connected to the electrostatic attractions and hydrogen bond, as indicated by pH and FTIR studies. The examination of MgO as a potential sorbent for other dyes (CV, MG, and MO) revealed the overall great potential and high selectivity of the MgO nanorods for cationic dye elimination in wastewaters.

## Figures and Tables

**Table 1:** The kinetic parameters for BF sorption on MgO nanoparticles.

Kinetics Model	Kinetic Equation	Parameter	Value
Pseudo-first-order [43]	$q_{t}={\;q}_{e}\left(1-{\;\exp}^{-K_{1.}t}\right)$	q_e_	495.9
		K_1_	1.62
		R^2^	0.80
Pseudo-second-order [47]	$q_{t}=\frac{k_{2}.q_{e}^{2}.t}{1+k_{2.}q_{e}.t}$	q_e_ (exp)	496.81
		q_e_ (cal)	496.34
		K_2_	0.0715
		R^2^	0.98
Liquid film diffusion [45]	$\ln\left(1-\left(\frac{\mathrm{qt}}{\mathrm{qe}}\right)\right)=-K_{\mathrm{LF}}*t$	K_LF_ (min^−1^)	5936
		R^2^	0.60
Intra-particle diffusion [48]	$q_{t}=k_{\mathrm{dif}}t^{1/2}+C$	K_dif1_ (mg/g min^0.5^)	1.496
		C_1_ (mg/g)	489.9
		R^2^	0.95
		K_dif1_ (mg/g min^0.5^)	0.057
		C_2_ (mg/g)	495.4
		R^2^	0.94
		K_dif3_ (mg/g min^0.5^)	0.010
		C_3_ (mg/g)	496.2
		R^2^	0.99

**Table 2:** Different isotherm constants for the adsorption of BF dyes onto MgO nanorods.

Model	Equation	Parameter	Value
Langmuir [52]	$\frac{C_{e}}{q_{e}}=\frac{1}{q_{m}K_{L}}+\frac{C_{e}}{q_{m}}$	q_m_ (mg/g)	493.90
		K_L_ (mg/g)	0.04
		R_L_ (L/mg)	0.17
		R^2^	0.985
Freundlich [53]	${\mathrm{lnq}}_{e}={\mathrm{lnK}}_{F}+\frac{1}{n}{\mathrm{lnC}}_{e}$	n	2.07
		K_F_ (L/mg)	176.21
		R^2^	0.999
Dubinin–Radushkevich [54]	${\mathrm{lnq}}_{e}={\mathrm{lnq}}_{m}-{K\varepsilon }^{2}$	q_m_ (mg/g)	504.70
		K × 10^−9^ (mol/Kj)^2^	2.04
		E (Kj/mol)	6.38
		R^2^	0.859
Temkin [55]	$q_{e}=\frac{\mathrm{RT}}{b}{\mathrm{lnK}}_{T}+\frac{\mathrm{RT}}{b}{\mathrm{lnC}}_{e}$	b (J/mol)	0.10
		K_T_ (L/mg)	2.63
		R^2^	0.921

**Table 3:** Comparison of the adsorption capabilities of MgO nanorods with various adsorbents.

Adsorbent	q_e_ (mg/g)	pH	References
Fe-MgO/kaolinite	10.36	10–12	[60]
Fe-ZSM-5 zeolite	251.87	5.0	[61]
Al/MCM-41	54.44	5	[62]
H_2_Ti_3_O_7_ titanate nanotubes	183.20	2.4	[63]
ESM	47.85	6	[36]
Starch-capped zinc selenide	222.72	7.0	[26]
Modified activated carbons	238.10	7–8	[11]
Loess clay (LC)	47.49	2–5.9	[64]
MgO	493.90	12	Current work

## Data Availability

The data presented in this study are available on request from the corresponding author.
